# The Development of Social Function Questionnaire for Chinese Older Adults

**DOI:** 10.3389/fpsyg.2022.794990

**Published:** 2022-03-24

**Authors:** Conghui Liu, Yunping Wang, Jing Li, Xinzhu Xing, Xiaojuan Chen, Jianbing Liu, Xuanna Wu

**Affiliations:** ^1^Department of Psychology, Renmin University of China, Beijing, China; ^2^Beijing Research Center of Urban System Engineering, Beijing, China; ^3^Department of Psychology, Capital Normal University, Beijing, China

**Keywords:** social function, Chinese older adults, questionnaire development, reliability, validity

## Abstract

**Objectives:**

Social function is an important indicator for physical and psychological health of older adults. However, there is a lack of a standardized questionnaire for measuring social function of older adults. This study developed a questionnaire to assess Chinese older adults’ social function.

**Methods:**

We used three samples (*N* = 2,257 aged ≥60 years) to test the reliability and validity of the questionnaire.

**Results:**

Based on exploratory and confirmatory factor analyses with two samples, the final version of Social Function Questionnaire for Chinese Older Adults (SFQCOA) contained three dimensions with 12 items: social support, social adaptation, and social engagement. Criterion validity test with the third sample showed that SFQCOA was positively related to the healthy indices and negatively related to the unhealthy indices.

**Conclusion:**

The validity and reliability of the questionnaire reach the requirements of psychometric standards, suggesting it is an effective tool for measuring social function of older adults.

## Introduction

The global population aged 60 or over numbered 962 million in 2017, more than twice as large as in 1980. The number of older persons is expected to double again by 2050 (United Nations, 2017). For China, the challenge for the increase of older adults’ population is quite big. By the end of 2020, China had 264 million people aged over 60 years, accounting for 18.7 percent of Chinese total population (National Bureau of Statistics of China, 2021). According to the United Nations (2019), the proportion of older adults’ population in China will account for 25.7% of the world’s older adult population by 2050.

The ever-increasing proportion of Chinese older adults has raised attention on people’s aging life. As people age, social function is vital for sustaining health and wellbeing while social dysfunction is significantly detrimental to health and wellbeing (Porcelli et al., 2019). In this study, we aimed to develop an effective scale to measure Chinese older adults’ social function.

Social function is the psychological and social resource that refers to an individual’s ability to interact with others in society and includes a broad range of social characteristics concerning social roles (Chen and Wang, 2015). Social function is an important indicator of the mental and physical health for older adults that influences disability and mortality rates in older adults (Holt-Lunstad et al., 2017).

According to the Social Production Functions theory (SPF, Lindenberg, 1989, 1996), older adults’ social function contains two dimensions: social support, and social engagement. Also, SPF-Successful Aging Theory (SPF-SA, Steverink et al., 1998) emphasized the importance of adaptation to physical, social, and psychological changes following the aging of older adults. Social adaptation was hypothesized to be another dimension of social function. Based on these theories, our study hypothesized that social function contains three dimensions: social support, social engagement, and social adaptation.

Social support means “the existence or availability of people on whom we can rely, people who let us know that they care about, value and love us” (Sarason et al., 1983, p. 127). Specifically, social support can be assessed by the extent that people achieve support from the familiar ones (del-Pino-Casado et al., 2018). Social support is an important component of social function because of its functional features of reducing psychological stress reaction, relieving mental tension, and improving social adaptability (Kotwal et al., 2016). The commonly used social support scales are: The Perceived Social Support Scale (PSSS) by Zimet et al. (1988), which assesses perceived availability and satisfaction with support received from family, friends, and so on. And, the Parent–Child Social Support Questionnaire by Wang et al. (2005), which measures the support both from the elders to young children and from young children to the elders.

Social engagement is a construct that broadly captures people’s involvement in social activities (Bourassa et al., 2017). Social engagement is usually measured by the tendency and the frequency of attendance on different kinds of social activities that facilitate social ties (Berkman et al., 2000; Kotwal et al., 2016). Commonly used social engagement scales for older adults are the Participation Scale by Van Brakel et al. (2006) and the Index for Social Engagement Scale (Mor et al., 1995).

Social adaptation refers to older adults’ flexibility to adapt to the environment and their changing roles (Bao et al., 2018). Lazarus and Folkman (1984) used coping as an important measurement of adaptation. The styles of coping are shaped by the adaptational context out of which it is generated (Folkman and Lazarus, 1988). In our research, social adaptation can be measured by how well people get along with changes (especially that come with aging). One Commonly used social adaptation scale is the Social Adaptation Self-evaluation Scale (SASS, Bosc et al., 1997).

To our knowledge, there is a paucity of standardized instruments for assessing social function for Chinese older adults. Most of the previous instruments had the following shortcomings. (1) They often measure isolated dimensions (e.g., social engagement/social support was used to describe social function, Sarason et al., 1987; Bourassa et al., 2017) rather than multiple and interrelated dimensions. (2) Those instruments lack psychometric properties, which means that they might not detect the psychological traits accurately. For example, Kotwal et al. (2016) considered multiple dimensions to assess social function. However, construct validity was not examined. Bao et al. (2018) developed a questionnaire of social health, but the reliability and validity were not satisfactory. (3) Those instruments were used to diagnose individuals with disabilities (e.g., Social Disability Screening Schedule; World Health Organization, 1980) rather than to focus on the social function for normal older adults. (4) Many previous measurements were developed under Western culture (e.g., Social-Adaptive Functioning Evaluation, Harvey et al., 1997). As many previous studies suggested, the measurement of social function for Chinese older adults may have different patterns from other cultures (Kim et al., 2008; Yang et al., 2020). For example, in Chinese culture, family support often plays a more vital role in social support (Leung et al., 2007).

The aim of this study is to develop a questionnaire with qualified reliability and validity that can effectively measure multiple aspects of social function of Chinese older adults. In this study, we explored the structure of social function and developed SFQCOA by exploratory factor analysis (EFA) and confirmatory factor analysis (CFA) on two samples of Chinese older adults. The three-factor structure of SFQCOA was hypothesized to be a convincing model for measuring social function for Chinese older adults. We also explored the correlations between SFQCOA and criterion variables based on data from a large sample. Based on previous studies (Eisele et al., 2012; Bourassa et al., 2017), we predicted that SFQCOA would be positively related to the positive criteria, such as happiness and wellbeing, whereas SFQCOA would be negatively related to the negative criteria, such as depression and the symptoms of dementia (Porcelli et al., 2019).

## Materials and Methods

Based on the previous relevant scales and questionnaires, we collected a list of items for measuring the social function of older adults. Considering our construct assumption, items were split into three themes: social support, social adaptation, and social engagement. We took three phases to develop our proposed scale: item development, scale development, and scale refinement.

### Phase 1. Item Development

#### Item Pool Development Procedure

Firstly, to build the initial item pool, we collected instruments that were related to social function (e.g., social domain question from Health Status Questionnaire, Radosevich and Pruitt, 1995; Social Functioning in Dementia scale, Sommerlad et al., 2017; Social-Adaptive Functioning Evaluation, Harvey et al., 1997; Index for Social Engagement, Mor et al., 1995; Short-Form Health Survey, SF-36, Ware and Sherbourne, 1992). Questionnaires measuring social support, social engagement, social adaptation, and other social domains of health and wellbeing were included in the initial item pool.

Secondly, 10 experts who are specialized in geriatric research and clinical work evaluated the effectiveness and appropriateness of items. According to their suggestions, the redundancy items were deleted and some items were modified to meet the reading habits of the older adults and to effectively reflect the social function status of the older people. Fifty-four items out of 120 items were excluded after experts’ evaluation. The reserved 66 items were refined and polished. Three themes were then identified for social function of Chinese older adults.

Thirdly, 10 older adults were recruited to give further advice about the appropriateness of the content and format of items so that all items can be easily understood by the elderly without ambiguity. These 10 participants (age over 60, ranging from 60 to 85) were recruited from a community in Haidian District, Beijing, China, whose education level ranged from primary school to graduate school. They read the whole scale and were asked to retell the meaning of each item. Sixty-six items were all reserved because they had appropriate statements and represented well for social function. Finally, the initial version of SFQCOA was formed by the 66 items: 30 items for social support, 20 items for social adaptation, and 16 items for social engagement. Each item is rated on a 4-point Likert-type scale (1. Disagree; 2. Somewhat disagree; 3. Somewhat agree; 4. Agree).

### Phase 2. Scale Development (Sample 1)

#### Item Analysis

##### Participants

According to the Constitution of China, citizens aged over 60 were older adults. As the border of 60 was widely used in many other studies (Zhang et al., 2020), we recruited older adults aged no less than 60 (ranging from 60 to 96, *M* = 73.61, SD = 8.47) in our study. Two hundred and sixty-five participants were recruited from 16 districts and counties in Beijing, China in November 2018. Participants were volunteered for the experiment and paid with gifts worth 20–30 yuan after the experiment. The demographic characteristics are shown in Table 1.

**Table 1:** Demographic characteristics of the study sample.

Demographic characteristics	Number of participants (%)			
	Sample 1 (*n* = 265)	Sample 2 (*n* = 278)	Sample 3 (*n* = 1,714)	Total (*N* = 2,257)
*Age*				
60–64	50 (18.8)	132 (47.5)	467 (27.2)	640 (28.3)
65–69	51 (19.2)	72 (25.9)	448 (26.1)	568 (25.2)
70–74	38 (14.3)	37 (13.3)	322 (18.8)	397 (17.6)
75–79	39 (14.7)	19 (6.8)	211 (12.3)	269 (11.9)
80–84	48 (18.1)	27 (9.7)	144 (8.4)	218 (9.7)
≥85	39 (14.7)	5 (1.8)	122 (7.2)	165 (7.3)
*Gender*				
Male	48 (18.1)	48 (17.3)	549 (32.0)	645 (28.6)
Female	217 (81.8)	230 (82.7)	1,165 (68.0)	1,612 (71.4)
*Marital status*				
Unmarried	1 (0.4)	3 (1.1)	22 (1.3)	26 (1.1)
Married	166 (62.6)	191 (68.7)	1,144 (66.7)	1,501 (66.5)
Divorced	17 (6.4)	20 (7.2)	84 (4.9)	121 (5.4)
Widowed	65 (24.5)	43 (15.4)	301 (17.6)	409 (18.1)
Unknown	16 (6.1)	21 (7.6)	163 (9.5)	200 (8.9)
*Education*				
Primary School or Below	20 (7.6)	28 (10.1)	423 (24.6)	471 (20.9)
Junior High school	54 (20.3)	92 (33.1)	504 (29.4)	650 (28.8)
Senior High School	70 (26.4)	101 (36.3)	459 (26.8)	630 (27.9)
Junior College	69 (26.1)	38 (13.7)	149 (8.7)	256 (11.4)
Bachelor	50 (18.8)	19 (6.8)	104 (6.1)	172 (7.6)
Master and above	2 (0.8)	–	4 (0.2)	7 (0.3)
Unknown	–	–	71 (4.1)	71 (3.1)

##### Procedure

After signing the informed consent forms, the participants were asked to fill the questionnaires, including SFQCOA and other criterion scales (for participants who were inconvenient to read or write, volunteers would read aloud the questionnaires for them and complete the questionnaires according to their oral responses).

##### Analysis

We calculated the correlations between items and the total score of the 66-item initial version of SFQCOA. Items with item-total correlation coefficients less than 0.35 were excluded (Anderson and Gerbing, 1988). After that, 48 items were reserved, including 22 items for social support, 14 for social adaptation, and 12 for social engagement.

#### Exploratory Factor Analysis

##### Analysis

After item analysis, we performed an EFA on sample 1 to explore the factor structure of the 48-item version of SFQCOA. EFA was conducted by SPSS 25.0.

##### Results

Firstly, the Kaiser–Meyer–Olkin (Kaiser, 1974) measure of sampling adequacy was 0.84, and the Bartlett sphericity test was used to establish whether there was a common factor among the total correlation matrix, *χ*^2^ = 4810.86, *df* = 1,128, *p* < 0.001, indicating that the correlation matrix was appropriate for factor analysis.

Secondly, based on our theoretical construction, we extracted three factors using the principal components analysis with varimax rotation. We eliminated the items with low factor loadings or high cross-factor loadings (Fabrigar et al., 1999), leaving 18 out of 48 items. Then, we conducted another EFA with the 18 items by maximum likelihood extraction with oblique rotation (promax). Eigenvalues of the three factors are 4.70, 2.61, and 1.75, accounting for 50.4% of the total variance. The three factors were identified as social support (seven items), social adaptation (six items), and social engagement (five items).

### Phase 2. Scale Development (Sample 2 and Sample 3)

#### Confirmatory Factor Analysis (Sample 2)

##### Participants

278 participants were recruited from 16 districts and counties in Beijing, China in March 2019. Participants were all aged over 60 (ranging from 60 to 88, *M* = 67.56, SD = 6.18). The demographic characteristics are shown in Table 1.

##### Analysis

In order to further explore the structural validity of the questionnaire, we used Amos 22.0 software to conduct confirmatory factor analysis on the questionnaire through maximum likelihood estimation. We used goodness-of-fit index (GFI), non-normed fit index (NNFI), comparative fit index (CFI), root mean square error of approximation (RMSEA), and standardized root mean square residual (SRMR) to assess the fit of our model (Bentler, 1992; Brown, 2006). Satisfactory fit cutoff criteria were based on CFI, GFI, and NNFI values higher than 0.90, RMSEA values close to, or less than 0.06, and SRMR close to, or less than 0.08 (Hooper et al., 2008).

##### Results

To determine whether the three-factor model had a better fit structure than the one-factor model, we conducted model comparison for the 17-item questionnaire (One item in this model was eliminated for low factor loading, and 18 items were reduced to 17 items). According to those indices, the three-factor model showed a better fit than the one-factor model (see Table 2). However, our results suggested that the 17-item model did not fit the data well. Therefore, we eliminated five items (leaving four items for each factor) that had relatively low factor loadings as well as seemed to be repeated measures for the corresponding three factors, and then we reanalyzed the model fit. After the trimming, the model fit became acceptable. Comparisons of the three models are shown in Table 2.

**Table 2:** Confirmatory factor analysis and model comparisons (Sample 2, *n* = 278).

	*χ^2^*	*df*	*χ^2^/df*	GFI	NNFI	CFI	RMSEA	SRMR
One-factor model (17-item)	735.71	119	6.18	0.71	0.41	0.48	0.14	0.13
Three-factor model (17-item)	259.06	116	2.23	0.90	0.86	0.88	0.06	0.06
Three-factor model (12-item)	97.06	51	1.90	0.94	0.92	0.94	0.06	0.05

#### Model Fit Across Genders (Sample 3)

##### Participants

1,714 participants were recruited from 16 districts and counties in Beijing, China in June 2019. Participants were all aged over 60 (ranging from 60 to 99, *M* = 70.22, SD = 8.07). The demographic characteristics are shown in Table 1.

##### Analysis

To test whether the 12-item SFQCOA behaved the same when measuring both genders, we separately performed confirmatory analyses for males and females in sample 3.

##### Results

Results suggested that both models fit the data well. Details see Table 3. The 12-item questionnaire and the factor loadings see Table 4. Inter-factor correlations of the three factors are 0.17 (*p* < 0.001) for social support and social adaptation, 0.38 (*p* < 0.05) for social support and social engagement, and 0.12 for social adaptation and social engagement.

**Table 3:** Model comparisons between genders (Sample 3, *n* = 1,714).

	*n*	*χ^2^/df*	GFI	NNFI	CFI	RMSEA	SRMR
Total sample	1,714	5.05	0.98	0.96	0.97	0.05	0.04
Male	549	2.69	0.96	0.94	0.96	0.06	0.05
Female	1,165	3.47	0.98	0.96	0.97	0.05	0.04

**Table 4:** Factor loadings for the 12-item model (*n* = 1,714).

Item	Factor loading		
	1	2	3
*Factor 1: Social support*			
I have a lot of family and friends to communicate with.	0.58		
Family or friends have given me a lot of help and support.	0.66		
When I meet with difficulties, I always have reliable friends to help me.	0.75		
My neighbors always care about me.	0.59		
*Factor 2: Social adaptation*			
Now the vacant life let me feel very boring.		0.67	
The changes in my role after retirement have not been easy for me to adapt to.		0.62	
I would be distressed that my position at home was not what it used to be.		0.64	
I often suffer because people around me do not treat me as they used to.		0.76	
*Factor 3: Social engagement*			
I’m interested in taking part in community activities.			0.80
I often take part in volunteer activities.			0.59
Participating in social activities makes me feel fulfilled and satisfied.			0.78
Taking part in activities makes me feel my own value.			0.80

Items of social adaptation are all scored reversely, because all the positive ones were excluded after exploratory and confirmatory analyses.

### Phase 3. Scale Refinement (Sample 3)

#### Criterion Validity

##### Criterion Scales

Participants from sample 3 completed a set of criterion scales.

The Ascertain Dementia 8 (AD8, Hughes et al., 1982; Li et al., 2012) contains eight items including eight ways indicating changes caused by cognitive problems in the last several years (e.g., less interest in hobbies/activities). The rating has three alternatives: Yes, a change; No, no changes, and N/A, do not know. The total AD8 score is generated by summing up the number of items responded with “yes, a change.” The score ranges from 0 to 8 (Galvin et al., 2005).

The Center for Epidemiologic Studies Depression Scale (CES-D, He et al., 2013) was used to measure Chinese older adults’ depression. It contains nine items (e.g., I feel depressed). The score of every item ranges from 1 to 10. CES-D showed great reliability and validity for Chinese adults.

The Mini-Mental State Examination (MMSE, Folstein et al., 1975) was a widely used screening test for cognitive impairment and dementia (Tombaugh and McIntyre, 1992). The total score of MMSE is generated from 30 questions (e.g., Make up and write a sentence about anything. This sentence must contain a noun and a verb), and scores from 0 to 30.

The 17-item Activity of Daily Living (ADL) was an instrument that was adapted from the scales by Lawton and Brody (1969). ADL measures older adults’ daily activities which include bathing, dressing, going to the toilet, shopping, etc. Participants were asked about difficulties with performing these activities. Responses were scored using a four-point scale: “can do it without difficulties,” “can do it but with difficulties,” “can do it with help,” and “cannot do.” The corresponding score assigned to each response ranged from 1 (high function) to 4 (low function).

The Index of Well-Being scale (IWB, Campbell et al., 1995) contains two subscales including general affective index (eight items, e.g., How do you feel about your life? Reversed scoring, rating from 1 to 7, happy to painful) and life satisfaction index (one item, how satisfied or dissatisfied are you with your life in general? Rating from 1 to 7, very dissatisfied to very satisfied). The total score ranges from 9 to 63.

The sit and reach test (Wang and Zhang, 2015) assesses the flexibility of body. The participants were asked to sit on the front part of a chair (42 cm high) with one foot straight, and then reach their hands toward their toes and stretch as far as they can. The distance between their fingertips and toes was recorded in centimeters. The distance values range from negative to positive, and the higher the value, the more flexible the participant is.

##### Results

The total score of SFQCOA had positive relations with healthy indices of physical and psychological health (including ADL, IWB, MMSE, and sit and reach distance), and negative relations with unhealthy indices (including AD8 and CES-D; see Table 4). For each factor, there was a consistent result that the correlation between MMSE/AD8 and social engagement reached a significant level. However, the correlations of MMSE/AD8 with social support and social adaptation did not reach a significant level (see Table 5).

**Table 5:** Correlations coefficients between social function and criterion variables (*n* = 1,714).

Measure	Social support	Social adaptation	Social engagement	Total score
ADL	0.17^***^	0.12^***^	0.34^***^	0.32^***^
IWB	0.30^***^	0.33^***^	0.16^***^	0.39^***^
MMSE	−0.01	0.01	0.28^***^	0.15^***^
AD8	−0.03	0.001	−0.22^***^	−0.13^***^
CES-D	−0.23^***^	−0.35^***^	−0.21^***^	−0.41^***^
Sit and reach (left foot) (cm)	0.15^***^	0.15^***^	0.26^***^	0.27^***^
Sit and reach (right foot) (cm)	0.15^***^	0.16^***^	0.26^***^	0.28^***^

MMSE, Mini-Mental State Examination; AD8, Ascertain Dementia 8; ADL, Activity of Daily Living; IWB, Index of Well-Being; CES-D, Center for Epidemiologic Studies Depression Scale.

*** *p* < 0.001.

#### Test–Retest Analysis (Sample 1)

To analyze the stability and consistency of the 12-item SFQCOA, 48 participants in sample 1 (*N*_male_ = 8; *M*_age_ = 70.1, SD = 8.2) were invited to complete SFQCOA questionnaire for a second time (February 2019, interval: about 3 months). The test–retest reliability of the overall scale—SFQCOA—was 0.72 (*p* < 0.001), and for the three factors—social support, social adaptation, and social engagement—were 0.51 (*p* < 0.01), 0.59 (*p* < 0.001) and 0.59 (*p* < 0.001), respectively.

#### Internal Consistency (Sample 1–3)

The values of internal consistency were shown in Table 6. Cronbach’s *α* coefficients of the total SFQCOA were 0.75 for sample 1, 0.68 for sample 2, and 0.75 for sample 3. Most of Cronbach’s *α* coefficients of the total and subscales were above 0.70, which indicates a good internal consistency.

**Table 6:** Cronbach’s *α* coefficients of three samples.

	Social support	Social adaptation	Social engagement	Total score
Sample 1	0.71	0.79	0.68	0.75
Sample 2	0.73	0.72	0.69	0.68
Sample 3	0.74	0.77	0.82	0.75

## Discussion

This study describes the development and validation of the three-factor (social support, social adaptation, and social engagement) structured questionnaire—SFQCOA, which to our knowledge, is the first to measure Chinese older adults’ social function from a comprehensive and systematic perspective. First, consistent with our hypothesis, we found that the three components of SFQCOA were consistent across two older adult samples. Second, the total scale and the subscales of the 12-item SFQCOA showed good internal consistency. Third, consistent with previous studies (Bourassa et al., 2017; Porcelli et al., 2019), the total and subscale scores of SFQCOA were positively correlated with positive criterion variables (ADL, IWB, and sit and reach distance) and negatively correlated with negative criterion variables (CES-D, AD8). In detail, older adults with better social functioning (including social adaptation, social engagement, and social support) would have higher activity levels, greater life satisfaction, better physical health, and more positive emotions.

However, we did not find the significant relationships between scores of MMSE and AD8 and social support, which is inconsistent with most of the previous studies (Lazarus and Folkman, 1984; Börsch-Supan and Schuth, 2014). One possible explanation is that the emotional component (such as the availability of family members and friends with whom to communicate) of social support might have no or little relationship with cognition (Eisele et al., 2012). In addition, inconsistent with previous studies (Social-Adaptive Functioning Evaluation, SAFE, Harvey et al., 1997), we did not find a significant relationship between the scores of MMSE and AD8 and social adaptation. One possible reason is that SAFE and SFQCOA measure different components: SAFE focuses on the mixture of social support, social engagement, and other factors, whereas SFQCOA only focuses on adaption. Another possible reason is that previous studies measuring social adaptation focused on the resilience of more severe stressful events (e.g., bereavement; Aşiret and Dutkun, 2018), while SFQCOA focuses on normal stressful events (e.g., retirement). Unlike social support and social adaptation, social engagement showed positive relations with cognitive function (positive relations with scores of MMSE and negative with AD8). The results demonstrate the complexities of the association between social function and older adults’ cognitive function.

Our study had several limitations. First, all three samples used Chinese older adults in Beijing, the capital of China. Compared to the elderly in other regions, the elderly that live in Beijing tend to enjoy better living conditions as well as have higher education levels, which might allow them to perform differently in social support, social adaptation, and social participation (Peng et al., 2021). Future research may enlarge the sampling range to confirm the validity of SFQCOA for older adults in other regions in China or in Western cultural backgrounds. Second, the sample was biased toward the female. However, males and females may perform differently in the three aspects of social function. For example, in the research by Ang (2018), men were illustrated to engage more in social activities than women. Therefore, future studies should recruit more gender-balanced samples. Third, we included broad-based samples rather than clinical samples. Clinical samples may work better for identifying cutoffs indicating a significant level of social function.

Despite the limitations, SFQCOA can be considered as an effective measurement to test the social function for Chinese older adults with good validity and reliability. Moreover, the results that social function is positively related to other mental and physical health may inspire a better understanding of older adults’ social lives and the aging society problems.

## Data Availability Statement

The raw data supporting the conclusions of this article will be made available by the authors, without undue reservation.

## Ethics Statement

The studies involving human participants were reviewed and approved by The Research Ethics Committee of Renmin University of China. The patients/participants provided their written informed consent to participate in this study.

## Author Contributions

CL, JLi, and JLiu had contributed on the conception and design of study, and on the acquisition of data. YW, XX, and XC had participated on the analysis of data. CL, YW, JLi, and XW had participated on the interpretation of data and drafted the manuscript. All authors contributed to the article and approved the submitted version.

## Funding

This work was supported by the Humanities and Social Sciences Foundation of the Ministry of Education of the People’s Republic of China under Grant (18YJA190007); Beijing Municipal Social Science Foundation under Grant (17GLC067); Mental Health Education Platform, Major Innovation & Planning Interdisciplinary Platform for the “Double-First Class” Initiative, Renmin University of China; Interdisciplinary Center for Philosophy and Cognitive Sciences Platform, Major Innovation & Planning Interdisciplinary Platform for the “Double-First Class” Initiative, Renmin University of China.

## Conflict of Interest

The authors declare that the research was conducted in the absence of any commercial or financial relationships that could be construed as a potential conflict of interest.

## Publisher’s Note

All claims expressed in this article are solely those of the authors and do not necessarily represent those of their affiliated organizations, or those of the publisher, the editors and the reviewers. Any product that may be evaluated in this article, or claim that may be made by its manufacturer, is not guaranteed or endorsed by the publisher.
